# The role and application prospects of plant-derived bioactive peptides in exercise fatigue recovery

**DOI:** 10.3389/fnut.2026.1767738

**Published:** 2026-03-27

**Authors:** Zhan Gao, Xiupeng Yin, Qinglei Cao

**Affiliations:** 1 Basketball Teaching and Research Office, Capital University of Physical Education and Sports, Beijing, China; 2 School of Ecology and Environment, Zhengzhou University, Zhengzhou, China; 3 Department of Physical Education, University of Science and Technology Beijing, Beijing, China

**Keywords:** anti-inflammatory, antioxidant, energy metabolism, exercise fatigue, plant-derived bioactive peptides, sports nutrition

## Abstract

Exercise-induced fatigue is a regular physiological event that impairs sports performance and prolongs recovery time. While conventional recovery methods exist, there is a pressing need for natural, effective, and sustainable alternatives. This review aims to comprehensively review the emerging role of plant-derived bioactive peptides (PBPs) in sports nutrition, highlighting their innovative potential compared to traditional supplements. We demonstrate that PBPs alleviate fatigue through multi-targeted remarkable mechanisms: scavenging reactive oxygen species (ROS) to mitigate oxidative stress, downregulating pro-inflammatory cytokines to protect muscle tissue, and activating specific metabolic pathways (e.g., AMPK) to accelerate glycogen resynthesis and optimize energy utilization. Consequently, the application of PBPs offers a compelling solution to delay fatigue and accelerate physical recovery. However, the field faces significant new challenges, including the lack of standardized dosages, unmapped structure-activity relationships, and a scarcity of human clinical trials. By addressing these unexplored aspects, this study provides a critical theoretical foundation and future directions for developing PBPs as next-generation functional ingredients in personalized sports nutrition.

## 1 Introduction

### 1.1 Background of exercise-induced fatigue

Exercise-induced fatigue constitutes an important issue to both athletes and fitness enthusiasts, since it not only hampers the improvement of sports performance but also slows down post-exercise recovery. This multifactorial physiological condition appears to involve several systems like oxidative stress, inflammation, and metabolisms (1). Therefore, the therapeutic cure of exercise-induced fatigue is an area of prime importance of research interest. Conventional recovery means like nutrition supply and physical exercise exhibit certain defects (2), which have inspired scientists to explore potential alternatives.

### 1.2 The potential of plant-derived bioactive peptides

Recently, plant-source bioactive peptides have received more interests as a potential alternative strategy for alleviating workout-induced fatigue and accelerating recovery, owing to their special bioactivities as well as the safety record. Plant peptide, especially the protein hydrolysate ones, possesses certain bioactivity such as antioxidant and anti-inflammation ability (3). The low-molecular-peptide has good bioavailability for human absorption, which is also characterized in low molecular weight. These have already been proven to be effective in dealing with oxidative stress through the scavenging of free radicals and by improving the endogenous antioxidant defense mechanisms (4) through reduction of free radical mediated cellular damage due to high intensity exercise. They also control inflammatory pathways (5) to reduce post exercise muscle soreness and fatigue, because of inflammatory reactions.

The exercise recovery benefits that plant peptides offer is numerous. They increase energy metabolism by means of improving the performance of mitochondria and increasing the usage of fatty acids (6) thus able to be used as an energy source during long physical activity. Besides, some of them are associated with muscle repair and regeneration, such as soybean and other legumes-derived bioactive peptides are found to stimulate muscle protein synthesis, promoting both muscle recovery and growth (7). By new progress of extraction and characterization technology (8), enzymatic hydrolysis and fermentation with microorganisms have been used to obtain bioactive peptides for specific functions (9). Introducing such peptides to nutrition supplements and functional food provides novel answers which are responding to the demands of athletes during recovery (10).

### 1.3 Knowledge gaps and review objectives

Overall, studies on the plant origin peptides as means to prevent exercise fatigue and as promoter for muscle recovery are expected to be advantageous approach, with their antioxidant and anti-inflammatory activities highlighted as valuable tools in the field of sports nutrition. However, future research for elucidating their exact mechanisms of actions and specific delivery systems is still essential for exploiting these discoveries into real-life applications.

To address these gaps, the present review is intended to provide an overview of the most recent developments of studies related to the influence of the plant based bioactive peptides (PBPs) on the accelerated post-exercise recovery, focusing on their bioactivities, mechanisms and applications. Therefore, the physiological mechanisms of exercise-induced fatigue are explained, and the antioxidant, anti-inflammatory and energy metabolism-regulation activities of PBPs are critically discussed. Finally, it identifies existing research gaps and proposes future directions for investigation, intending to provide a theoretical foundation and scientific reference for developing PBPs as natural and effective functional ingredients in sports nutrition and recovery products.

## 2 Literature search strategy

To provide a comprehensive narrative overview of the role of plant-derived bioactive peptides (PBPs) in exercise fatigue recovery, a thorough literature search was conducted. This approach was chosen to synthesize the current mechanisms, applications, and prospects of PBPs in sports nutrition.

### 2.1 Search strategy and databases

The literature search was performed across multiple mainstream academic databases, including PubMed, Web of Science, Scopus, ScienceDirect, and CNKI. The search covered publications from 2015 to 2025, with the final search updated on (Insert Last Search Date, e.g., 28 February 2026) to ensure the inclusion of the most recent advancements. The core search strategy utilized a combination of Boolean logic (AND/OR) with the following keywords: (“plant-derived bioactive peptides” OR “plant protein hydrolysates”) AND (“exercise fatigue” OR “sports nutrition” OR “muscle recovery”) AND (“antioxidant” OR “anti-inflammatory” OR “energy metabolism”).

### 2.2 Study selection criteria

To maintain the scientific rigor of this narrative review, specific inclusion and exclusion criteria were established. Studies were included if they met the following criteria: (1) original peer-reviewed research articles focusing on the biological activity and mechanisms of PBPs in exercise fatigue recovery; (2) studies with clear experimental designs, including *in vivo* animal models, human clinical trials, or *in vitro* cell experiments; and (3) literature presenting complete quantitative data and physiological outcomes.

Conversely, the exclusion criteria comprised: review articles, conference abstracts, and non-peer-reviewed materials. Literature published in languages other than English or Chinese, as well as studies with ambiguous experimental designs or incomplete data, were also excluded.

### 2.3 Data extraction

Two independent authors screened the literature based on titles, abstracts, and full texts. The extracted data, including research models, specific peptide types, intervention dosages, targeted mechanisms, and quantitative physiological indicators—were cross-checked to ensure accuracy and reliability. Any discrepancies during the screening and extraction process were resolved through discussion and consensus with a third author.

## 3 Physiological mechanisms of exercise-induced fatigue

### 3.1 Oxidative stress and free radical production

**FIGURE 1:**
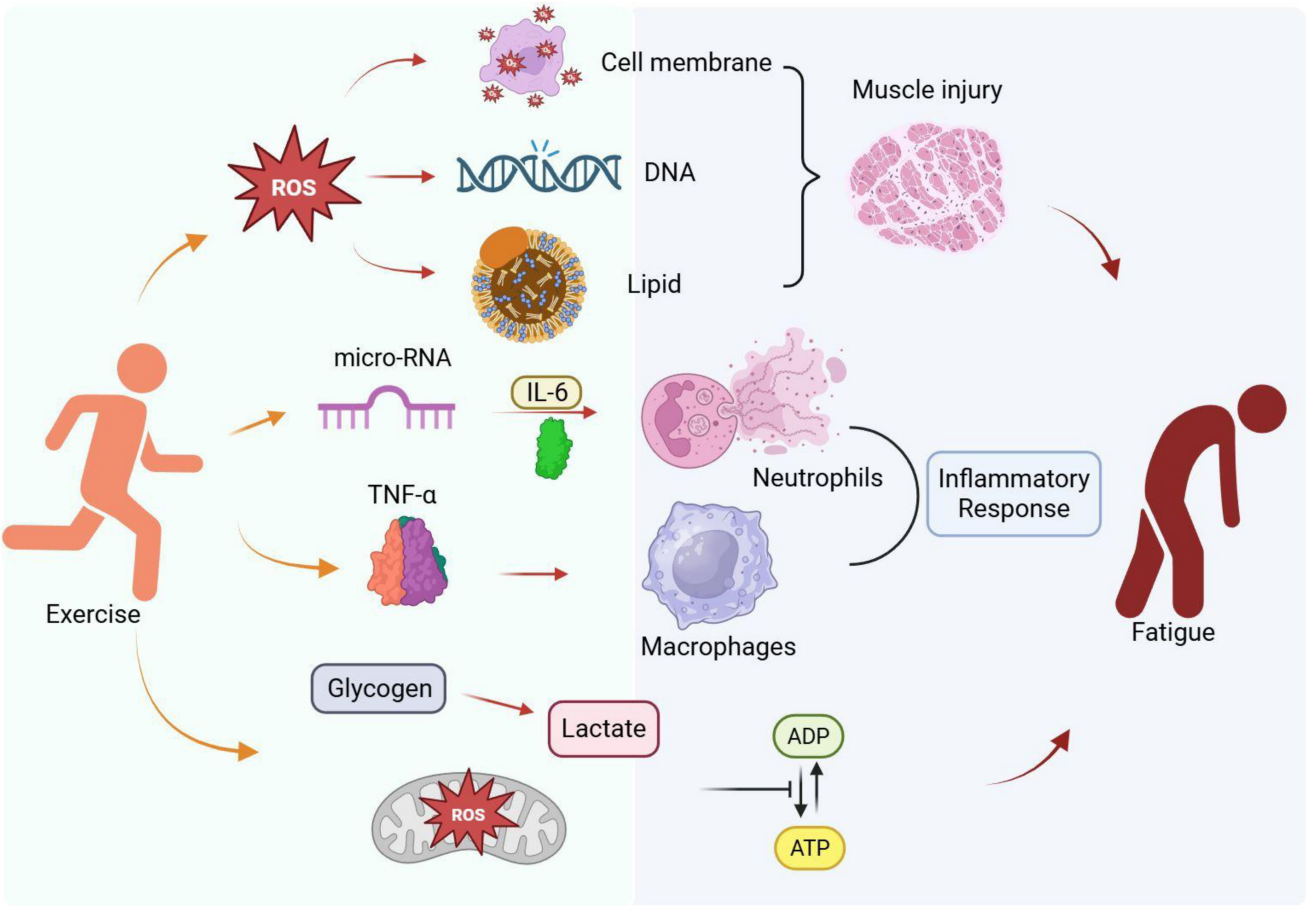
Physiological mechanisms of exercise-induced fatigue.

This means that high-intensity exercise induces a substantial generation of Reactive oxygen species (ROS). During intense physical activity, the body significantly increases its oxygen consumption to meet heightened energy demands, which simultaneously drives the overproduction of Reactive Oxygen Species (ROS). Under normal physiological conditions, ROS are tightly regulated and serve essential roles in cell signaling and homeostasis. However, during high-intensity exercise, the generation of ROS rapidly exceeds the scavenging capacity of the body’s endogenous antioxidant defense system. This imbalance triggers oxidative stress. Consequently, excessive ROS act as harmful molecules that inflict severe oxidative damage on critical cellular structures—including lipids, proteins, and DNA—ultimately leading to mitochondrial dysfunction and cellular injury (11, 12). Muscle fibers are especially sensitive to these effects because free radicals may lead to damage of the cell’s membrane and inhibit recovery following workouts (Figure 1).

Free radical attack on the membranes of cells leads to peroxidation of lipids which is damaging for both the membrane structure and function. Also, proteins can be damaged by free radicals—their structure and function may be altered, and it may influence enzymatic activity in addition to leading to disturbances in intracellular signaling processes. Damage to DNA caused by free radicals can lead to mutation and genomic instability; the cumulative effects over periods of time could increase the risk of cancer (13, 14). Studies have shown oxidative stress markers like malondialdehyde (MDA), 8-hydroxydeoxyguanosine (8-OHdG) rise remarkably after a strenuous exercise (15, 16), which does mean the exercise has aggravated oxidative damage.

Oxidative stress does not only lead to the direct death of cells, it can also have longer-term effects on human performance, such as an increase in fatigue in athletes, performance decrement, and delay of muscle recovery and regeneration. Therefore, studies of antioxidant supplementation have become an area of continued interest. Exogenous and endogenous antioxidants have the potential, either scavenging free radicals or damaging free radicals, respectively, to minimize damage and accelerate recovery (17, 18). Nevertheless, there has been controversy on its effectiveness, since increased consumption could destabilize the balance of reactive oxygen species (ROS) which is beneficial for regular physiological processes like the immune response or to muscular adaptation to training (16, 19).

### 3.2 Inflammatory response and muscle injury

High intensity exercise-induced mechanical damage to skeletal muscle triggers a local inflammatory response, which is essential for repair and regeneration (20). Muscular injury during the post-exercise period leads the repair process through microtears in the muscle fibers (14), the induction of multiple inflammatory response pathways, and the release of DAMPs which can alert the adaptive immune system through the recruitment of immune effector cells like neutrophils and macrophages (18, 19). The presence and the behavior of these cells has two sides: on one side they are required to remove the debris and launch the repair process, but, on the other side, they may excessively activate and increase the damage. This comes from a contrast between the cytokines acting on the pro and anti-inflammatory sides, for example: Interleukin-6 (IL-6) and tumor necrosis factor-alpha (TNF-α) are secreted in major amounts after a trauma. These cytokines expressed at heightened levels can stimulate neutrophil infiltration that is required for immediate repair, though if left unchecked can induce further trauma to the muscle.

The initial immune responder at the site of injury, the neutrophil releases reactive oxygen species (ROS) and proteases on stimulation. These molecules aid clearance of the damaged tissue but can also induce harm to healthy surrounding tissue (21). A prolonged inflammatory response can lead to a harmful cascade that causes continual muscle fiber damage and delayed healing (22–24), but possibly also chronic inflammation in the muscles which can induce atrophy (25). Additionally, the timing and extent of the inflammatory phase play a major role in recovery processes: the acute inflammatory phase supports muscle reparation whereas an extended inflammation phase deteriorates recovery processes and further destroys muscle fiber. For instance, microRNA-223-3p (miR-223-3p) has been shown to control inflammation during muscular recovery by suppressing the pro-inflammatory mediator interleukin 6 (IL-6) and thus creating a pro-reparative environment (26).

As shown, muscle injury evoked by exercise is a complex response including multiple cells and multiple molecules (Figure 1). Systematic knowledge of the complex dynamic changes of exercise induced muscle injury, especially the roles played by each important cytokine or immune cell, is critical to formulate optimal recovery plans and to ameliorate the undesirable consequences induced by muscle injury. We recommend further investigation on therapeutic targets capable of influencing the inflammatory environment that could promote optimal muscle regeneration, and at the same time avoid the threat of persistent inflammatory response and muscle atrophy (27, 28).

### 3.3 Energy metabolism disorders

Metabolism disorders caused by energy supply/metabolism metabolic disorders play crucial roles in generation of exercise fatigue among athletes and performers under strenuous physical activities (Figure 1), of which, the main feature is imbalance between ATP (adenosine triphosphate) consumption and synthesis with decline of ATP consumed during exercise, resulting in failure of muscle activities. However, this is not all; glycogen depletion exacerbates this imbalance because glycogen, a critical reservoir of energy, is extensively used during extensive or high-intensity exercise. Glycogen depletion itself leads to the reduction of glucose for its use in the synthesis of ATP plus lactate accumulation, which induced metabolic acidosis (26). With the increase of lactate, muscle cells decrease their pH, which hampers muscle cell contraction and regeneration (29). This state presents a pattern of complaints and symptoms of muscle exhaustion, loss of performance, and delayed recovery (29, 30) all highly impairing to athletic performance (31).

Mitochondrial dysfunction will lead to abnormality in energy metabolism processes. Mitochondria is called as the “powerhouse” in a cell and synthesizes ATP for cells by oxidation phosphorylation process. If mitochondrial function is decreased because of the reason of insufficient oxygen supply, or ROS caused damage (31, 32), the efficiency of ATP synthesis decreases significantly. The result will be energy deficiency and the inability of recover after the exercise. Thus, the vicious cycle induced by ATP depletion, glycogen depletion and mitochondria dysfunction will evidently shorten athletes’ performance, reducing its recovery.

Physiologically, the specific type of countermeasures are based on active recovery, i.e., low-intensity effort after training, which can improve the blood circulation and clear lactate (33); similarly, rational nutritional plans based, i.e., on post-exercise addition of carbohydrates aimed at replenishing glycogen store and addition of proteins aimed at reparation muscle, effectively avoid the perturbances of energy metabolism (34). Further studies need to explore the interdependencies between energy metabolism, training effort and forms of recovery to determine better strategies for designing training plans to enhance athletic performance (35, 36).

## 4 The source and classification of plant-derived bioactive peptides

### 4.1 Overview of bioactive peptide sources and the unique advantages of PBPs

The therapeutic and health-promoting effects of bioactive peptides have garnered immense attention across various natural sources. Recent advancements have highlighted the diverse applicability of non-plant peptides; for instance, marine-derived peptides from red seaweed (*Pyropia vietnamensis*) exhibit novel bioactive characteristics, while animal-derived peptides like hydrolyzed collagen from various tissues are widely applied for their tissue-repairing properties (37, 38). Furthermore, dairy-derived interventions, such as the anti-diabetic potential of casein and the bioactive peptides generated in probiotic-cultured yogurt, have demonstrated significant metabolic and sensory benefits (39, 40). However, while these diverse sources are invaluable, plant-derived bioactive peptides (PBPs) offer distinct, highly sustainable, and hypoallergenic advantages specifically tailored for sports nutrition. Compared to animal or marine sources, PBPs uniquely combine potent antioxidant, anti-inflammatory, and energy-modulating effects with a lower environmental footprint, making them a critical frontier in exercise fatigue recovery.

### 4.2 Soy peptides: rich in hydrophobic amino acids with antioxidant and anti-inflammatory activities

**FIGURE 2:**
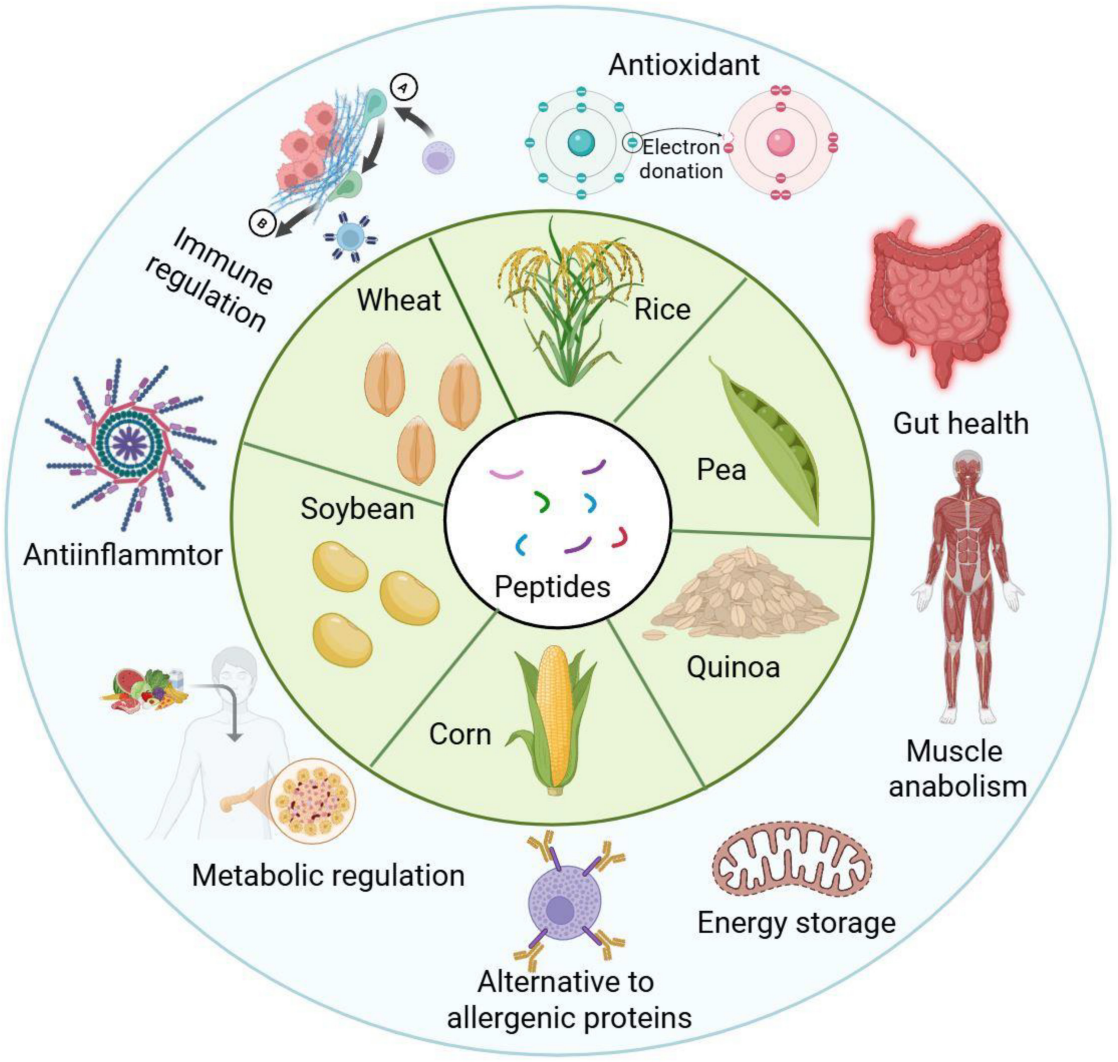
Bioactivities of plant-derived bioactive peptides (PBPs) its function.

Soy protein peptides originated from hydrolysate of soy proteins could possess the characteristic of antioxidant and anti-inflammatory effects firstly based on their huge contents of hydrophobic amino acids (Figure 2), with which various bioactivities in the alleviation for exercise-induced fatigue and recovery have been deeply investigated in recent decades. They reflect their antioxidant potential by scavenging free radicals and reducing oxidative stress, which plays a crucial role in maintaining cellular integrity in exercise. Soybean peptide’s amino acid composition of leucine, isoleucine, and valine can modulate up regulation of endogenous antioxidant enzymes and control redox signaling pathways as antioxidant defense mechanism (36).

On the other hand, the anti-inflammatory actions of soybean peptides are also important, since they could help to decrease the production of pro-inflammatory cytokines and inflammation-related markers, under several different physiological states. In athletes perform heavy exercise, exercise-induced inflammation and oxidative stress compromise both recovery and performance. Soybean peptides have been demonstrated to lower levels of inflammatory mediators (TNF-α, IL-6) in macrophages and other immune cells (31) as natural anti-inflammatory agents.

Furthermore, soybean peptides may also modulate metabolism including lipid metabolism and glucose metabolism (32) that are important for exercise fatigues recovery and general health. Dietary soybean peptide intake could enhance the recovery process from an acute bout of exercise but also be potentially significant for long-term health by mitigating the effects of oxidative stress or inflammation. Such health claims require validation in future clinical trials exploring the mechanisms, thus providing a basis for using them in functional foods and dietary supplements (41).

### 4.3 Wheat peptides: glutamine-containing peptides promoting immune regulation and gut health

In fact, the wheat peptides, particularly the rich in glutamine ones are worthy to be used to provide benefit to the immune-regulation and gut health (Figure 2), especially glutamine is an energy supplier to the intestinal epithelial cells, which is the fundamental support to preserve the function and barrier of gut. It has been shown that the wheat peptide could be able to induce immune regulation, which is extremely valued as a valuable property among many functional foods working toward healthy purposes. For example, a systematic review pointed out that some plant protein components, such as wheat protein, exert a positive impact on the consumers’ immune status by the release of bioactive peptides during enzymatic degradation (42).

In fact, wheat peptides have demonstrated to have a role in promoting production of immunoglobulins and other mediators of the immune system, acting as immunomodulatory agents that could avoid occurrence of infections and inflammatory pathologies. In addition, they constitute relevant immune adjuvants and have been shown to influence gut microbiota composition: regular intake of wheat peptides ameliorates the composition of gut microbiota and helps alleviating symptoms associated with constipation (43) and indicating capabilities to augment the gastrointestinal motility of the intestine, as well as general health of the gut. It is well-known that a healthy gut microbial composition correlates with enhanced immune system activity and lower gut-associated diseases incidence. Moreover, as the presence of bioactive molecules as dietary fibers and phenolic acids in wheat, are also implicated in the health of intestinal microbiota by stimulation of the good gut flora development and as well, in the generation of anti-inflammatory SCFA production (44).

Immune regulation and gut integrity are dual functionality of wheat peptide which will make it very important element in dietary interventions toward better health and to manage chronic diseases. With advancement in the understanding of bio-mechanisms of wheat peptides, coming years research should focus on identification of the pathway that wheat peptides initiate to perform this functionality to develop more novel functional foods utilizing this functionality.

### 4.4 Corn peptides: high in branched-chain amino acids for promoting protein synthesis

Corn peptides (CPs) are rich in branched chain amino acids (BCAAs) as leucine that is important for the promotion of muscle protein synthesis and can help alleviate exercise-induced fatigue, improve exercise recovery, enhance its function to regulate lipid metabolism, and antioxidant activity (45). Such properties are beneficial for the athlete and people involved in the high intensity physical activity. Animal studies have confirmed the beneficial impact of CPs on performance during exercise. In mouse studies performed on exhaustive swimming test, CPs increased the swimming capacity, also reduced the rate of increase in body weight during exercise (45), suggesting possible use for endurance enhancement and recuperation. In addition, the values of post-exercise blood urea nitrogen (BUN) – an index reflecting the excessive muscle protein breakdown – were lowered dramatically in murine models of exhaustive swimming following continuous CP supplementation (typically administered at doses ranging from 200 to 800 mg/kg/day for 4 to 8 weeks), thereby potentially inducing reduction in muscle catabolism and facilitating recovery (Figure 2).

Moreover, under these specific intervention protocols, CPs increase glycogen reserves in muscles as well as the liver which are essential to supply energy during endurance exercises and ensure that athletes get the benefit of an adequate amount of muscles glycogen for further training and competitions. The mechanisms described above imply the activation of the signaling pathways characteristic for muscle metabolism: CPs raise the expression of the proteins: AMP-activated protein kinase (AMPK), peroxisome proliferator-activated receptor gamma coactivator 1-alpha (PGC-1α) and phosphoinositide 3-kinase (PI3K) (45), which control the metabolism and the biogenesis of mitochondria. Further, Akt which is phosphorylated down-stream PI3K also plays a role in anabolic pathways which are required for muscle healing and hypertrophy.

In addition, CPs might also influence the composition of gut microbiota, and correlation analyses suggest that there were up-regulated beneficial bacteria (such as *Lactobacillus* and *Akkermansia*) upon supplementation with CP (45). This regulation of gut health promotes enhanced nutrient uptake and is thought to reduce inflammation which contributes further to recovery from exercise-induced fatigue. Going forward, CPs will increasingly serve a core role in performance enhancing, recovery-focused dietary plans for athletes and the general public.

### 4.5 Pea peptides: low allergenicity for special populations

Pea peptides have lately become a good choice for those with certain diet restrictions mainly because they have low allergenicity (Figure 2). Some recent research has also revealed that the allergenicity of pea protein can be greatly reduced by enzymatic hydrolysis (46), and consequently for those who suffer from food allergies or intolerances. An indirect ELISA and RBL-2H3 cell assay were used for the evaluation of allergenicity of pea peptides (46). It was demonstrated that when mice were sensitized to pea protein, a higher level of total IgG1 and IgE antibodies against peptide in the mice following exposure to pea protein. Pea proteins treated by enzymatic hydrolysis could significantly decrease the allergenicity of pea protein hydrolysates and even ultrafiltration fraction F1 (also known as F1), pure fraction F1-2 and F1-2 hydrolysis (46).

The defined peptide (ADLYNPR), extracted from fraction F1-2, was able to bind weakly to targeted IgE and IgG1 (46), and showed an EC50 of 6.63 ng/mL, almost 36.83 times higher than the EC50 of the isolated pea protein (46). This demonstrates that pea peptides can be hypoallergenic and can be considered safe for children with allergies or people who cannot eat legume because of intolerance.

Furthermore, besides allergenicity, the pea peptidic composition has well-recognized health benefits supporting the recovery from exercise-induced fatigue. Peas are a good source of essential amino acids. They help the recovery in muscle repair and recovery; therefore, by including pea protein into nutrition regiment following exercise can support recovery and overall wellbeing and performance in exercise, especially in people facing diet restrictions. While pea peptides can compete allergenic protein sources (whey and soy) with the attraction from market side, due to increasing demand of plant sources proteins, more opportunities are created for food industry and nutritionist side to develop and use hypoallergenic pea peptides for major uses like sport nutrition and dietary supplementation.

### 4.6 Other plant peptides: emerging sources such as rice peptides and quinoa peptides

The peptides obtained from plant products such as rice and quinoa have shown their valuable potential in nutrition and therapy recently (Table 1). Among the products, the hydrolysis of rice protein (rice peptides) results in bioactivity of the bioactive rice peptides including antioxidant, antihypertensive, and anti-inflammatory activity. Example is peptides from rice bran protein demonstrate strong ACE inhibitory property (47) that reveals their application for controlling hypertension, their radical-scavenging antioxidant properties might contribute to the cure of diseases resulting from oxidative stress (48).

**TABLE 1:** Classification, key characteristics, and bioactivities of plant-derived bioactive peptides (PBPs).

Plant-derived peptide type	Key amino acid composition/features	Core bioactivities in exercise fatigue recovery	Target molecules/pathways	Quantitative/qualitative evidence	References
Soy peptides	High in hydrophobic amino acids (leucine, isoleucine, valine)	1. Antioxidant: Scavenges free radicals; upregulates endogenous antioxidant enzymes 2. Anti-inflammatory: Inhibits pro-inflammatory cytokine production 3. Metabolic regulation: Improves lipid and glucose metabolism	- Redox signaling pathways - Inflammatory pathways (e.g., in macrophages) - Lipid/glucose metabolic cascades	- Reduces levels of tumor necrosis factor-α (TNF-α) and interleukin-6 (IL-6) in immune cells - Enhances activity of antioxidant enzymes	(31, 32, 36, 41)
Wheat peptides	Rich in glutamine; coexists with dietary fibers and phenolic acids	1. Immune regulation: promotes immunoglobulin synthesis 2. Gut health: modulates microbiota; alleviates constipation 3. Synergistic gut protection	- Enzymatic digestion-dependent immune regulatory pathways - Gastrointestinal motility-related signaling - Short-chain fatty acid (SCFA) synthesis pathways	- Improves gut microbiota profile; reduces constipation symptoms - Promotes growth of beneficial bacteria	(42–44)
Corn peptides (CPs)	High in branched-chain amino acids (BCAAs), especially leucine	1. Muscle anabolism: stimulates muscle protein synthesis 2. Anti-fatigue: prolongs endurance; reduces muscle catabolism 3. Energy storage: increases glycogen reserves in muscle/liver	- AMP-activated protein kinase (AMPK) - Peroxisome proliferator-activated receptor gamma coactivator 1-alpha (PGC-1α) - Phosphoinositide 3-kinase (PI3K)/Akt pathway	- Prolongs exhaustive swimming time in mice - Reduces post-exercise blood urea nitrogen (BUN) levels by 15%–20% - Increases glycogen content in muscle tissue	(45)
Pea peptides	Low allergenicity; rich in essential amino acids	1. Hypoallergenicity: safe for allergy-prone populations 2. Muscle repair: supports post-exercise muscle regeneration 3. Alternative to allergenic proteins (whey/soy)	- Immunoglobulin E (IgE)/immunoglobulin G1 (IgG1) binding pathways - Muscle protein synthesis cascades	- Ultrafiltration fraction F1-2 shows EC*50* (6.63 ng/mL) 36.83 × higher than native pea protein (reduced antibody binding) - No detectable allergenic reactions in sensitized mice	(46)
Rice and quinoa peptides	Rice: contains Val-Tyr-Thr-Pro-Gly; quinoa: all essential amino acids	1. Antioxidant: scavenges reactive oxygen species (ROS) 2. Metabolic regulation: inhibits glucose-metabolizing enzymes (quinoa) 3. Immunomodulation: enhances immune response (quinoa)	- Angiotensin-converting enzyme (ACE) inhibitory pathways (rice) - Glucose metabolism-related enzyme cascades (quinoa) - Immune regulatory signaling	- Rice bran peptides exhibit IC*50* of 0.8–1.2 mg/mL for ACE inhibition - Quinoa peptides inhibit α-amylase/α-glucosidase activity - Rice peptide Val-Tyr-Thr-Pro-Gly improves glucose metabolism	(21–23, 47–49)

The progress in extraction and characterization methods has made it easier for us to identify certain bioactive sequences of peptides. In fact, recent emerging peptide Val-Tyr-Thr-Pro-Gly was confirmed to have potentially beneficial roles in cognitive ability (49) and in blood glucose regulation and hence presenting diverse health benefits from rice-derived peptides.

Like soy and wheat, quinoa with its high nutritional value also provides high bioactive peptides. Quinoa protein hydrolysates have great antioxidant and anti-inflammatory benefits (21) and can have potential use in chronic diseases prevention, such as diabetes and cardiovascular diseases. Peptides from quinoa have been demonstrated to be bioactive compounds against the enzymes that take part in the glucose pathway (48). This feature can bring about advantages in function of food development targeting glucose regulation and controlling diabetes. Additionally, since quinoa is a source of all essential amino-acid (22) and therefore is sustainable dietary protein source (21, 49), other applications such as use in food formulations can be envisaged. According to (23), Quinoa peptides show immunomodulatory activities which can also be expected to be linked to immune modulation.

The studies on rice and quinoa peptides are an active research area and will be beneficial to nutrition and health. With recent technological improvements in extraction/characterization procedures, specific bioactive peptides have been detected paving the way for their utilization in functional food products as a nutraceutical. With further elucidation of their modes of action, these peptides will be expected to increasingly play important roles in diet strategies intended to improve health and help control chronic diseases (Table 1).

## 5 Antioxidant effect of plant-derived bioactive peptides

### 5.1 Direct scavenging of free radicals

The immediate scavenging of free radicals is a primary mechanism through which plant-based bioactive peptides alleviate exercise-induced fatigue and accelerate recovery. Peptides rich in hydrophobic amino acids, such as tyrosine (Tyr) and phenylalanine (Phe), can effectively donate electrons to neutralize free radicals, thereby mitigating oxidative stress-mediated damage (30, 31). By donating the electron, these low-affinity peptide stabilized the high-reactive free radicals and thus preventing the destruction of the cells. Furthermore, their hydrophobic nature enhances their interaction with cellular membranes, providing a direct shield against ROS produced during physical activity. This activity can be carried out in another way besides their own effect that the plant-derived peptides also inhibit oxidative stress through metal ions chelation. Metal ions such as iron and copper catalyze the production of hydroxyl radicals from hydrogen peroxide via the Fenton reaction, which exacerbates free radical generation. These increase the biosynthesis of free radicals and by being chelated to the metal ions, the bioavailability of the metal ions is reduced leading to a decrease in potentially damaging radicals (29, 32).

Such a dual action – both via direct free radical quenching, and via chelating metal ions – acts simultaneously as both direct shield against ROS-induced oxidative cellular damage and to support post-exercise repair. Antioxidant efficacy is moreover linked to modulation of the immune response in that the peptides are able to modulate cytokine profiles (e.g., pro-inflammatory cytokines, other recovery mediators, etc.) (34, 36). Further investigations are needed to unravel exact sequences of peptides and protein structures associated with an antioxidant functionality and looking to their implementation for sport food and training programs in order to take full advantages for their amelioration of athletes’ fitness.

### 5.2 Enhancement of the endogenous antioxidant system

Another critical anti-fatigue mechanism of PBPs is the enhancement of the body’s endogenous antioxidant defense system. One of the important strategies is to upregulate the expression and activities of some important antioxidant enzymes, such as superoxide dismutase (SOD), catalase (CAT) and glutathione peroxidase (GSH-Px). The above enzymes are involved in counteracting the generated ROS when exercising: SOD by converting superoxide anions to hydrogen peroxide and then this hydrogen peroxide is broken down into water and oxygen by CAT thus reducing the oxidative damage (24).

Evidence indicates that dietary supplementation with PBPs significantly augments these enzymatic activities (24, 25). Moreover, plant peptides promote the synthesis of glutathione (GSH), a crucial non-enzymatic radical scavenger in the intracellular redox cycle (50). Peptides containing GSH precursors, such as cysteine, are particularly effective at boosting GSH levels and reinforcing overall antioxidant capacity (50, 51).

Antioxidants: exercise promotes consumption and availability: the presence of exercise also means that there is an increased need for antioxidants, as regular physical activity augments the expression of nuclear factor erythroid 2-related factor 2 (Nrf2) (25), a transcription factor that is associated with activation of the antioxidant response element (ARE) in order to induce the expression of a number of antioxidant enzymes, namely SOD, CAT and GSH-Px. Also, plant-based dietary antioxidants can increase this effect and make sure that the bodies antioxidant system helps the body recover from damage and become more capable. Endogenous antioxidants serve to regulate oxidative damage to achieve redox homeostasis, necessary to carry out an effective cell signaling and an overall metabolic efficiency. ROS levels that are too high cause damage and fatigue but are essential to initiate recovery and enable more effectiveness of the bodies(52). Furthermore, modulation of glutathione (GSH) does detoxify toxic metabolites besides their role in cell signaling modulation of cell-signaling and inflammatory pathways and muscle recovery (51). Consequently, it is expected for a continued investigation into mechanisms of how plant-derived peptide modulates antioxidant pathways to expand their potential in sport nutrition and recovery.

### 5.3 Protection of mitochondrial function

Mitochondria are the primary sites of cellular energy production; thus, maintaining their functional integrity is crucial for delaying the onset of exercise-induced fatigue. PBPs demonstrate significant potential in protecting mitochondria during the post-exercise recovery period. One major protective mechanism is the stabilization of the mitochondrial membrane potential, which is essential for ATP synthesis. Studies show that specific plant peptides can prevent the loss of membrane potential typically caused by oxidative stress and cellular exhaustion during intense workouts (30). Preserving this membrane potential ensures sustained ATP production and reduces the risk of mitochondria-mediated apoptosis, thereby accelerating energy restoration. Therefore, restoring the membrane potential is important in restoring energy during post-exercise while ensuring survival. Mitochondrially-induced cell apoptosis suppression is also carried out by plant derived peptides that reduce the recovery process resulting cell death with reduced loss of muscle cells as well as other tissues critical to manage fatigue (29). In addition, these peptides regulate ROS generation: their antioxidant effects nullify an excess of ROS maintaining the mitochondrial structure and function (31). A high production of ROS can damage mitochondria lipid, protein and DNA–which can be followed by mitochondrial pathologies as apoptosis.

Additionally, plant-based peptides have been demonstrated to stimulate mitochondrial biogenesis via a route involving activation of PGC-1α (32). PGC-1α increases the number of mitochondria present and has an enhanced capacity, thus aiding the cells in generating more energy and positively aiding recovery from exercise. This benefit from plant peptidic nature as a positive impact on the metabolic processes in mitochondria has demonstrated that these peptides have the ability to not only remove fatigue symptoms, but may also improve physical activity performance and physical conditioning capacity, which will increase the body’s resistance to subsequent exercise stimuli causing a setback such as overtraining and declining performance with practice periods (33). Further work will need to be conducted to understand how exactly these peptides work, to tap into these molecules for use in recovery programs for athletes, as well as those suffering from fatigue-induced disorders.

## 6 Anti-inflammatory effect of plant-derived bioactive peptides

### 6.1 Regulation of inflammatory factor expression

Plant-derived bioactive peptides could regulate expression of inflammatory factors as a mechanism of alleviating exercise-induced fatigue and enhancing recovery, among which the nuclear factor kappa B (NF-κB) signaling pathway is a main target. NF-κB is an important factor participating in the inflammatory response, so that plant-based peptide inhibits NF-κB activation, causing the expression levels of pro-inflammatory cytokines TNF-α and IL-6 to decrease (30, 31). A consequent increase in these pro-inflammatory cytokine levels after strenuous exercise or acute physical effort, are thus related to inflammation occurring along with skeletal muscular damage, which delay tiredness recovery. In this regard, the anti-inflammatory bioactivities shown by plant peptides act also as regeneration due to their suppression of NF-κB activity. Such all-in-one strategy demonstrates that they may contribute to the remedies for exercise-induced fatigue and exercise-recovery enhancement due to its quelling of transient inflammation response and stimulating of sustainable physiological readjustment that can be leveraged for the enhancement of exercises as well as anti-exercise fatigues (Table 2).

**TABLE 2:** Core mechanisms of plant-derived bioactive peptides (PBPs) in alleviating exercise-induced fatigue.

Functional category	Core mechanism	Target molecules/structures	Specific biological actions	Measurable physiological outcomes	References
Antioxidant effects	1. Direct free radical scavenging 2. Metal ion chelation 3. Endogenous antioxidant system activation 4. Mitochondrial protection	- ROS (superoxide anion, hydroxyl radical) - Iron (Fe^2^ + ), copper (Cu^2^ + ) - Superoxide dismutase (SOD), catalase (CAT), glutathione peroxidase (GSH-Px) - Mitochondrial membrane potential; PGC-1α	- Donates electrons to neutralize ROS (via tyrosine/phenylalanine) - Binds Fe^2^ + /Cu^2^ + to inhibit Fenton reaction - Upregulates SOD/CAT/GSH-Px activity; promotes glutathione (GSH) synthesis - Stabilizes mitochondrial membrane; inhibits mitochondrial apoptosis	- Reduces malondialdehyde (MDA) levels by 20%–30% post-exercise - Increases GSH content in muscle tissue by 15%–25% - Maintains mitochondrial ATP synthesis efficiency (>80% of resting state) - Lowers 8-hydroxydeoxyguanosine (8-OHdG) (DNA damage marker)	(24, 25, 29–32, 50, 51)
Anti-inflammatory effects	1. Inflammatory pathway inhibition 2. Anti-inflammatory mediator promotion 3. Muscle tissue protection	- Nuclear factor kappa B (NF-κB); mitogen-activated protein kinases (MAPKs) - Interleukin-10 (IL-10); nuclear factor erythroid 2-related factor 2 (Nrf2)/heme oxygenase-1 (HO-1) - Neutrophils; creatine kinase (CK); lactate dehydrogenase (LDH)	- Inhibits NF-κB activation to reduce TNF-α/IL-6 expression - Stimulates IL-10 secretion; activates Nrf2/HO-1 pathway - Reduces neutrophil infiltration; decreases CK/LDH leakage	- Lowers IL-6 levels by 25%–35% post-high-intensity exercise - Reduces delayed onset muscle soreness (DOMS) score by 1.5–2.0 points (10-point scale) - Decreases serum CK activity by 30%–40% within 24 h post-exercise	(30, 31, 34, 53, 55)
Energy metabolism regulation	1. Glycogen resynthesis 2. Lactate metabolism improvement 3. Fatty acid utilization optimization	- AMPK; glucose transporter type 4 (GLUT4); glycogen synthase - Lactate dehydrogenase (LDH); NADH/NAD + - Peroxisome proliferator-activated receptor alpha (PPARα); fatty acid β-oxidation enzymes	- Activates AMPK to promote GLUT4 translocation; enhances glycogen synthase activity - Increases LDH activity; modulates NADH/NAD + balance - Upregulates PPARα to promote fatty acid β-oxidation	- Accelerates muscle glycogen resynthesis by 40%–50% within 4 h post-exercise - Reduces blood lactate levels by 30%–35% post-exercise - Increases fatty acid oxidation rate by 20%–25% during prolonged exercise	(30, 31, 36, 57–62)

The future use of plant peptides in clinical and sports is increasing, as clinical studies validate their potential to influence inflammatory signaling and underline their safety and efficacy as components of dietary supplements. Further research is needed to clarify their mechanisms of action in relation with cell signaling pathways and prove the effectiveness of such actions on diverse populations in a clinical trial to consolidate their position in modern nutritional and medical approaches (36, 53).

### 6.2 Promotion of anti-inflammatory mediator production

In addition to suppressing pro-inflammatory cytokines, PBPs actively promote the secretion of anti-inflammatory mediators, such as interleukin-10 (IL-10), to facilitate exercise recovery. IL-10 is a crucial cytokine that maintains immune homeostasis. Several studies have established that specific plant-derived peptides can induce signaling cascades that upregulate IL-10 production (31, 53, 54). This upregulation effectively counteracts exercise-induced inflammation, resulting in attenuated muscle soreness, reduced fatigue, and an optimized environment for muscle injury repair.

Plant-origin bioactive peptides further provoke activation of Nrf2/HO-1 (nuclear factor erythroid 2-related factor 2/heme oxygenase-1) pathway. Nrf2 is a primary transcription factor involved in oxidation-reduction stress, and inactivation of Nrf2 can decrease both apoptosis and carcinogenesis. Nrf2 induces gene expression of the cytoprotective genes, such as HO-1. HO-1 is a regulator of anti-inflammatory with the function of pro-inflammatory mediator breakdown (34, 53). Through this pathway, plant-based peptides increase our body’s capacity to defend against post-exercise oxidative stress and the inflammation that causes it, combating exhaustion along with illness.

Finally, it seems that synergic association between IL-10 production and the Nrf2/HO-1 axis produces more effects which are potentially positive health effects on the cells, which may serve them as good functional components for recovery diets in athletes and fitness personnel. It is recommended to explore more mechanisms of plant peptides to be more applicable in functional foods and dietetic supplements (55, 56).

### 6.3 Protection of muscle tissue

The anti-fatigue recovers effect of plant-derived bioactive peptides on muscle have been widely focused (Table 2). An important underlying mechanism is that it can restrain neutrophil influx to muscle; too much neutrophil in muscle after intense exercise will lead to intensified muscle damage. Peptides of legumes and grains have been reported to possess anti-inflammatory activity (31, 36) that through modulation of the inflammatory pathways limit the recruitment of neutrophils to injury sites. Peptides of legumes and grains also stimulate the expression of anti-inflammatory cytokines along with reduction in the expression of pro-inflammatory mediators thus enhancing the microenvironment favorable to the recovery of muscle. Additional plant-derived peptides are helpful in lowering concentrations of muscle-enzyme leakage, for instance, significantly decreasing serum creatine-kinase (CK) activity within 24 h post-exercise (6, 11). Across various preclinical models, the oral administration of PBPs (with effective dosage ranges broadly reported between 0.5 and 1.5 g/kg/day depending on the botanical source) has consistently demonstrated the ability to curb the leakage of this critical muscle damage marker. Peptides rich in branched-chain amino acids (BCAAs) are particularly effective at diminishing post-exercise enzyme leakage (30, 31). This protective effect is likely achieved by stimulating muscle-protein synthesis and reducing oxidative stress, which collectively preserve muscle-cell integrity and functionality. In addition, antioxidant activities of plant protein peptides are also essential for muscle preservation. During the process of vigorous physical activities, there is a variety of oxidative injury caused to the cell tissue, which can hinder repair process and fortunately peptide extracts of soy, quinoa or other proteins can act as free radical scavengers (31, 36), in which free radicals damage can be reduced for muscle fibers structure preservation and tissue repair. Additional studies on specific peptide sequence and their modes of action are required in order to fully reach out the potential of the peptides used in sports nutrition as muscle health supporters (31, 36).

## 7 Regulation of energy metabolism by plant-derived bioactive peptides

### 7.1 Resynthesis

Recovery from exercise requires a replenishment of glycogen stores, with dietary bioactive peptides contributing to the repletion of the glycogen stores to ease exercise fatigue. One key process that this repletion of glycogen facilitates involves the AMPK pathway. AMPK is a conserved and powerful energy sensor that plays a major role in regulating cellular energy homeostasis (57). In skeletal muscle, AMPK activation has been demonstrated to enhance glucose uptake by facilitating the translocation of glucose transporter 4 (GLUT4) to the cell membrane. This mechanism stimulates glucose uptake to restore glycogen, depleted with exercise and maintains blood glucose concentration at homeostatic levels post-exercise accelerating muscle glycogen resynthesis during the acute post-exercise recovery window (e.g., within 4 h). This rapid resynthesis is particularly pronounced when specific PBPs are co-ingested with carbohydrates in endurance models, optimizing both the insulinogenic response and substrate availability. Additionally, plant-originated peptides also enhance glycogen synthase—a glycogen chain synthesizing enzyme. After strenuous exercise, glycogen synthase activity frequently becomes an ergogenic bottleneck of muscle glycogen synthesis; augmentation of the activity of this enzyme accelerates muscle recovery from glycogen depletion, and thus offsets muscle glycogen depletion-related exercise fatigue (36).

The synergic action is reached with the carbohydrates plant-derived peptides co-administered with the carbohydrate: on the one hand, the carbohydrates supply the needed substrates of glucose, while inducing insulin secretion by insulin action, activation of glycogen synthase and blocking of glycogen phosphorylase, on the other, plant-derived peptides can also further promote the insulin sensitivity for the sake of enhancing efficiency for glycogen storage (58). Post-workout time frame is a beneficial time-frame for intake of peptides due to high glucose and insulin sensitivity of the muscles, allowing increased synthesis of glycogen (30). In addition, plant peptide can control the expression of genes related to glycogen metabolism (31), modulating the transcriptional activity of major enzymes in glycogen synthesis that allows to increase the muscle glycogen storage. Regulatory through mTOR (mammalian target of rapamycin) pathway: this kind of regulation may be modulated, as it happens for mTOR, after a post-exercise recovery, but which has fundamental implication in both muscle development and glycogen resynthesis (63).

### 7.2 Improvement of lactate metabolism

Enhancing lactate metabolism plays a pivotal role in relieving exercise-related fatigue and optimizing sport performance and endurances and can mainly be accomplished by the activity augmentation of lactate dehydrogenase (LDH), the enzyme that facilitates the interconversion of lactate to pyruvate (and further) into the tricarboxylic acid (TCA) cycle for the generation of metabolic energy. Peptides, specifically of plant origin, have been demonstrated to potentiate LDH activity (31, 36) to facilitate efficient use of lactate as a fuel during endurance exercise and to maintain energetic performance over longer periods. Furthermore, these peptides could potentiate mitochondrial biogenesis gene expression resulting in enhancement of mitochondrial respiratory function and lactate oxidation in muscles.

The regeneration of pyruvate from lactate depends on cofactor availability (such as NADH, NAD+) and the mitochondrial condition of muscle cells. Possible plant-derived peptides may influence the cellular NADH/NAD + ratio (32, 33) and are likely to favor LDH-catalyzed pathways acting toward lactate removal, muscle fatigue, and pain prevention and acid-base balance even during intense training activities—to avoid muscle failures caused by acidosis (11, 12, 64). They also increase the pace of lactate metabolism both during and following exercise, through a more effective activation of mitochondria and higher oxidative phosphorylation rates (59, 60), leading to faster recuperation time and less fatigue, a feature especially helpful for athletes involved in any kind of long distance sport. Their mechanism of action deserves further evaluation in future research work and the outcome of their effectiveness be validated through clinical trials before they could be widely applied in sports nutrition (31, 36).

### 7.3 Optimization of fatty acid utilization

Fatty acid metabolism and using this readily available carbon source efficiently for maximizing energy source are essential for optimizing energy supply and recovery, especially with physical exercise (27). One of the favorable consequences from using plant bioactive peptides in animal and human diets is to activate peroxisome proliferator activated receptor alpha (PPARα) by up-regulating transcription factors of PPARα, which can help regulate the conversion of fatty acid during treatment (28). Activation of PPAR$\alpha$ enhances the fatty-acid $\beta$ -oxidation (31, 61), thus transforming them into energy needed for demand of activities during exercise, using stored fats and decreasing dependence on glycogen stores, as well as probably enhancing endurance.

In addition, efficient fat usage prevents accumulated fat post-exercise; as described above, fat concentration increases momentarily after intense exercise because of changes in hormones and energy recovery. Plant-derived peptides, with a PPARα stimulation effect, promote fatty acid oxidation instead of storage (53, 62), helping athletes to sustain a proper body composition, but also optimizing their metabolic flexibility between carbohydrate and fat use for different levels of exercise intensity and time duration. Finally, the antioxidant character of plant-derived peptides (60, 65) serves to limit exercise-induced oxidative stress contributing to their recovery.

## 8 Research on the application of bioactive peptides derived from food sources

### 8.1 Animal experimental evidence

**FIGURE 3:**
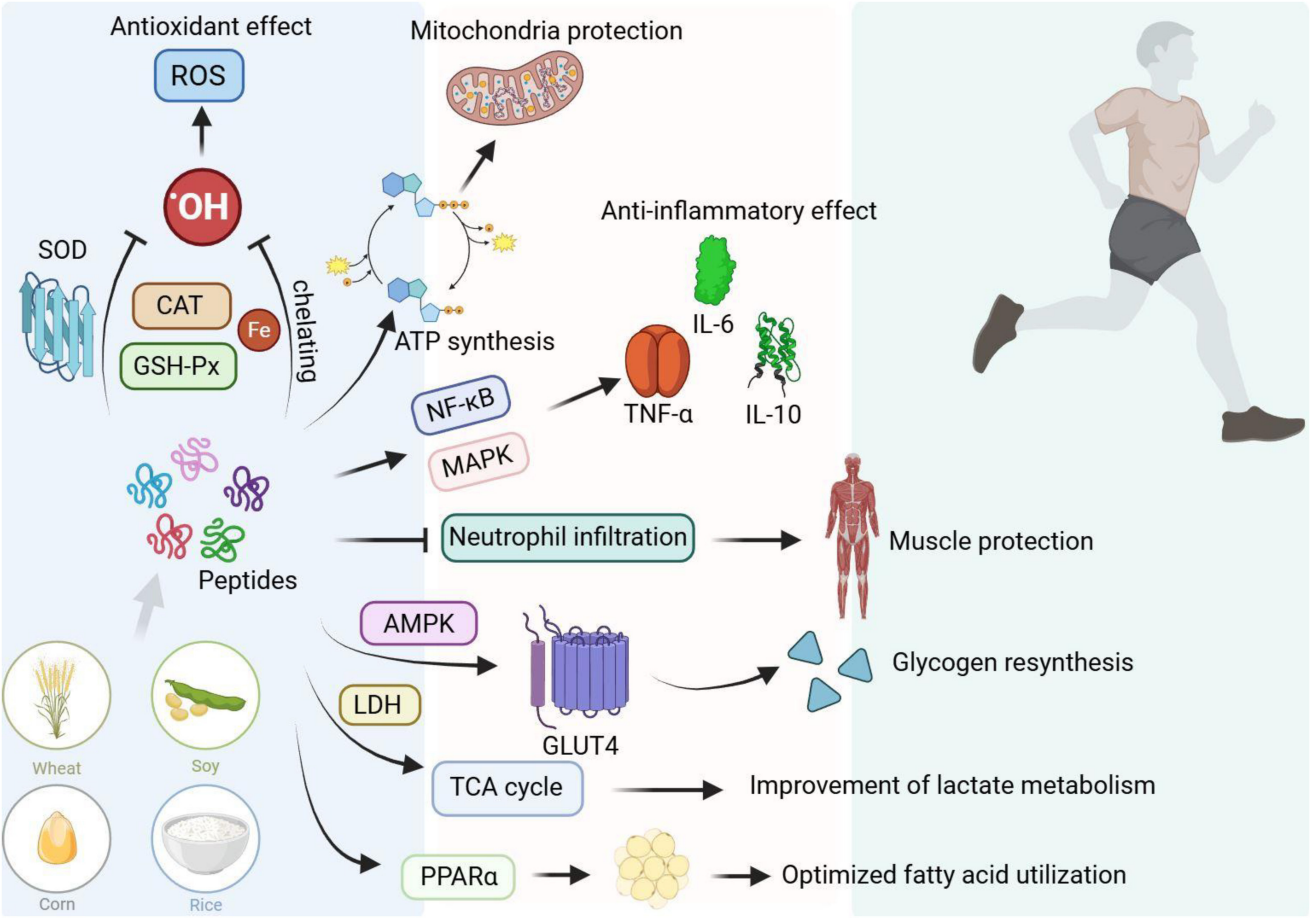
The mechanism of plant-derived bioactive peptides (PBPs) anti-fatigue.

Further studies are required to explore mechanisms of action, thereby increasing the scope for their wider applications in sports nutrition; helping athletes as successful intervention aimed at improving an athlete’s performance and health. It is evident from the results of studies done using animal models (specifically, the models of rats suffering from fatigue due to exercise) as promising and encouraging results about the beneficial applications of plant-derived peptides in reducing fatigue and enhancing the recovery process. It has also been established that supplementation with individual plant-derived peptide can indeed be more valuable in reducing fatigued related parameters including exercise abilities, metabolic parameters etc. For example, it has been found that supplementing rats with peptide could increase their time to exhaustion in the forced swimming test (32) and therefore, better endurance primarily due to modulating essential metabolic pathways of body with higher metabolism utilization (Figure 3).

The post-exercise blood lactate levels detected in rat models subjected to exhaustive swimming or treadmill tests showed that acute or short-term peptide supplementation (e.g., at dosages of 200–600 mg/kg/day) was able to greatly reduce lactate accumulation when measured immediately post-exercise (31). The effective ranges reported across these diverse studies highlight that while individual magnitude of reduction varies based on peptide concentration, the overall metabolic benefit remains highly consistent. The study is also in line with the prediction that these peptides stimulate skeletal muscle metabolic readjustment, with the purpose of enhancing production of energy. The plant-based peptides act through a range of different pathways (including the control of oxidative stress and inflammation), some types of bioactive peptides act antioxidant in function (61), interfering with reducing the oxidative injury of muscle tissue during maximal exercise. Moreover, it could also affect genes expression related to muscle repair and regeneration thereby facilitating recovery. It would be future interest to explore the lasting impact of supplementation with the aim of transferring findings from the laboratory into potential practical approaches to aid athletes and gym users (33).

### 8.2 Human clinical trials

The essential confirmations of the performance and contribution of plant-protein-derived peptide to exercise fatigue and recovery demand Human clinical investigations. At present, supplementation with peptides has been proven to speed up post-exercise heart rate recovery in athletes (66), which plays important role for cardiovascular fitness and the recovery of capacity. Fast heart rate recovery decreases training interval and optimizes training efficiency and capacity. Plant peptides have also proven to ease DOMS, a frequent reality that follows high intensity training (67). Clinical studies have suggested that athletes that were supplied with a peptide cocktail, reported diminished muscular soreness that consequently resulted in higher levels of subjective wellbeing, allowing them to continue with their normal training capacity (amount and intensity) despite a compensatory loss of performance linked to DOMS. It is probable that such effect should be associated to the anti-inflammatory activity of these peptides whose action is to regulate inflammation in favor of muscle repair (Figure 3).

For performance improvement, numerous clinical studies were reported showing performances of athletes—e.g., strength, endurance, and exercise capacity—in athletes consuming plant peptides. Additionally, substantial clinical evidence supports the potential of plant-based proteins in promoting muscle health. A 2021 systematic review and meta-analysis indicated that plant proteins showed no significant difference from animal proteins in enhancing lean body mass gain and muscle strength (68). For example, athletes who supplemented certain bioactive peptides showed enhanced power output and higher endurance during the high-intensity interval training; improvement can be attributed to greater muscle protein synthesis, down-regulation of oxidative stress and faster recovery that facilitate the increase in training loads without an increased risk of overtraining. Besides, in clinical experiments, higher recovery efficiency in peptide supplementers has been also observed (69), as it will cause less muscular fatigue after exercising and accelerate the pace of the athlete’s energy restoration—factors essential to keep up with hard training and race preparation for athletes. Better recovery will facilitate both athletes’ performance and mental state, as less tired athletes are better able to perform better during a game. Future studies could seek to determine mechanisms of action, ideal dosing/timing of peptide supplementation and the effects of long-term supplementation and application to other sports and populations (Figure 3).

### 8.3 Current status of product development

Finally, we observe that the use of plant-derived peptides in sports nutrition is spreading fast nowadays, in consonance with the general rise in consumer preferences related to natural functional compounds and with the confirmation, by research studies, of the actual potency of such food compounds that can effectively contribute to sport performance. In addition, peptides isolated from soybean and other legumes have been shown to be effective in stimulating repair of muscle tissue during exercise and counteract damage due to exercise-induced oxidative stress, being a desirable feature to include in a sports nutrition product (2–4), which is consistent with a trend of consumer demand for foods of plant origin (5) and an increasing knowledge of benefits relating the use of plant ingredients to health (6, 7). The interactions with other foods, particularly carbohydrates have also been considered for plant-peptides. The combination of protein or peptide with carbohydrates produced superior muscle glycogen resynthesis and MPS (i.e., recovery) and was found to be crucial for athletes recovering after efforts or other sorts of aggressive efforts (64). Certain peptide–carbohydrate formulations have demonstrated the ability to mitigate muscle soreness as well as overall muscle performance leading to better performance of the product.

Progress with extraction and purification has allowed the derivation of pure peptides with desirable nutritional effects by enhancing bioavailability, and therapeutic activity of nutritionally derived supplements. Strict quality control and regulation have meant that safe and beneficial products can be offered for consumer use. Nonetheless, practical issues remain in broad usability: peptide content per plant source may vary according to the extraction process used; health claims may require enhanced clinical evidence; and consumers have low awareness about this newly growing plant-peptide market, so continuous learning and advertising efforts will be needed for market expansion. As a conclusion, bioactive peptides isolated from plants have several potential application opportunities in the field of sports nutrition and from the currently available evidence they can be considered as effective and safe agents and with synergistic properties with other nutrients and with technological methods they should constitute part of the development of new sports nutrition products. To release their potential, currently remaining challenges and increasing knowledge and information to the consumer will become essential.

## 9 Prospects and limitations

### 9.1 The optimal dosage and timing of intake have not yet been standardized

Even though plant-based bioactive peptides seem to have potential for counteracting exercise induced fatigue, developing standardization in terms of an optimal supplementation dose and timing remain unaddressed issues. Research available in the literature suggests that effectiveness of peptides depends on the type of peptides, status of individual physiology as well as timing, prior to exercise (31, 60). The published effective dosages seem different, ranging from micrograms to grams, the reasons for which might be multifactorial and associated with the origin of the peptides and their bioactivity. This is true also as regards the time when supplementation is administered (pre-exercise, exercise or post-exercise supplementation). In fact, the effective doses of supplementation generally associated with maximum performance improvement, associated with delayed onset of fatigue, are observed and proved better with pre-exercise supplementation (70). It can thus be considered that post exercise supplementation favors the phase of recovery and muscle repair (70).

Even this benchmark is clouded because there will always be individuality (i.e., age, sex, fitness) and setting out guidelines that will guarantee the establishment of an individual supplementation plan seems even more difficult because RCT would be necessary to determine the impacts of different amounts and timing of supplementation on recovery performance. Complementary to this, technological systems such as metabolomics and proteomics will also have to contribute to the quest for understanding the mechanisms of action (65, 71), and standardization.

### 9.2 Need for in-depth research on structure-activity relationships

Profound investigation and analysis on the SAR of the bioactive peptides from plants are necessary for translational conversion and application based on biological activity. Amino acid composition, sequence-specificity and three-dimensional conformation, for example whether peptides are more hydrophobic or charged at certain positions, are all coupled to peptide function (41, 60). Nevertheless, existing literature concerning the connection between the structural characteristics and bioactivity are sporadic and this calls for comprehensive SAR analysis to help derive “ideal” peptide structures with respect to their future therapeutic use.

The application of analytical methods, including mass spectrometry, has dramatically accelerated the rate at which bioactive peptides can be found from diverse botanical origin (31, 70), though difficulties remain in understanding the physicochemical and biological interactions of peptides in a biological milieu. A thorough understanding of SAR will be critical to future therapeutic design by guiding us in structurally modifying a peptide (e.g., via added amino acids and chain length) which may stabilize, enhance bioavailability, and increase therapeutic benefit (62, 71). Molecular docking and molecular dynamics simulations are computational tools that provide insight into peptide-target interactions which facilitate the design of peptide therapeutics. SAR studies in food science and nutrition build on for example the development of functional foods rich in bioactive peptides that exert a specific health function, such as antihypertensive or antidiabetic (72, 73). A broader use of SAR is associated with the development of bioactive peptides; several bioactive peptides are susceptible to degradation in gastrointestinal tract limiting their oral bioavailability (16). It will enable us to design enzymatic resistant peptides with retained bioactivity during digestion (74, 75).

### 9.3 Long-term safety and metabolic pathways remain unclear

Finally, plant derived bioactive peptides seem to have application potential for sports medicine and fatigue recovery, yet many knowledge gaps remain surrounding their long-term safety and metabolism that stymie their greater use. Safety in the long-term is important to those who are taking supplements or functional foods for prolonged periods in sensitive people or those already compromised due to other health issues. There is a need for better description of potential adverse outcomes of consumption over extended periods of time. We note also lack of knowledge about non-identified biochemical pathways that brings questions about bioavailability as well as potential interactions with the rest of the diet or drugs. Differences in production might impact on the quality of peptidic entities, including purity and biological potency, motivating a production standardization for quality control. Moreover, allergenicity or toxicity hazards of such peptides from low-prevalence plants should be evaluated.

These knowledge gaps demand long term clinical trials that assess both safety and efficacy across a variety of populations and provide key information regarding long term effects as well as metabolic implications to inform rational use in dietary supplements and functional foods. Omics technologies, such as metabolomics and proteomics, will enable the detailed profiling of metabolic pathways as well as the discovery of biomarkers associated with efficacy and safety. Future work should unravel the biological pathways underlying peptide action to then identify specific metabolic pathways to which interventions for EIF recovery may target. Overall, although there are many possibilities of utilizing plant-based bioactive peptide for performance enhancement and recovery in sports, it should be noted that it is of crucial importance that the safety profiles as well as metabolic behaviors of these compounds under regular, repeated dietary applications should be well-defined via considerable systematic studies and clinical trials.

### 9.4 Demand for personalized supplementation regimens

While increasing appreciation for inter-individual variation in physiological response to exercise-induced fatigue (32, 36, 55) has highlighted the need for personalized supplementation regimens. Plant derived bioactive peptides effectiveness is fundamentally dependent on amino acid composition and structure, which differs inter-individual genetics, metabolism, and lifestyle variables (31, 60). Personalized regimens for optimizing recovery and performance need to factor in these differences. We see websites like Multi-Functional Plant Peptide Database (MFPPDB) (33) that can help personalize nutrition as we could select the peptides in accordance with our biological response to physical work through data on various metabolic pathways. Other technologies to aid personalization are nutrigenomics that capture nutritional genetics studies to pinpoint predispositions that can provide clues to nutrient metabolism and responses to supplements. The future studies will further identify, verify the effectiveness of the peptides among different population, develop best clinical standard operating procedures for application of peptides in a personalized approach and clinical trials for establishing its health effects (62, 70). The proposed development of tailored supplementation schemes, including plant based bioactive peptides, can be considered a suitable “frontier” in the field of sports nutrition with great potential to promote the wellbeing and performance of humans involved in physical activity.

### 9.5 Exploration and evaluation of novel plant sources

Searching and examining a potential new plant source of bioactive peptides is still an important direction of health and nutrition related investigation. Plant bioactive peptides were mainly extracted from dicotyledonous plants (31), and their actions are various. Recent developments in extractive, purificatory and identification technologies contribute to identifying new peptides with therapeutic potential. Web databases (such as MFPPDB (33), that contain the information of 1,482,409 therapeutic peptides from 121 different plant species listed by function (antibacterial, anticancer, etc.) are relevant for identifying new peptides because they provide standardized access of abundant number of peptides. Plant derived peptides are very structurally diverse with a special emphasis on cyclic ones which have been focused on in recent years due to their stability and bioactivity (41). As an example, cyclic peptides from BURP domain has a distinctive biosynthetic process which enriches its drug properties as an alternative pharmaceutical compound, providing novel approach toward untreatable diseases. Plant peptides, too, show an ability to modulate immune and anti-inflammatory response (76), attesting its utility in preventing non-communicable diseases and disease management.

Their involvement in regulating metabolism moreover brings their therapeutic potential, e.g., peptides from wheat gluten known for shielding yeast from osmotic stress (70) suggest potential preventive application against stress related diseases. Directing the lab studies toward applications in industry raises barriers such as involved extraction/purification techniques, expensive production methods and preclinical as well as clinical trials of considerable length (31). Finally, developing new resources of bioactive peptide from plants is an emerging vibrant research area that holds great promise to benefit human’s health and nutrition. With ongoing improvements of research and knowledge concerning bioactive peptide, a joint effort by scientists, the industries and authorities should be needed to implement it in practice to produce safe, efficient products based on peptides in the near future by bringing together different state-of-the-art techniques and databases in finding new opportunities in food, health and medical sectors.

### 9.6 Limitations of current data and research methodology

Despite the promising application prospects of PBPs in sports nutrition, the current body of evidence is constrained by several methodological limitations that require critical evaluation. First, there is a distinct lack of large-scale, well-controlled human clinical trials; much of the existing data is derived from *in vitro* assays or animal models, which cannot fully replicate human physiological responses to exercise. Second, as previously noted, the absence of standardized dosage and timing protocols makes it difficult to formulate specific dietary guidelines. Third, substantial inter-individual variations in effects—driven by genetics, baseline fitness, and metabolism—are often underreported or not comprehensively analyzed in current studies. Consequently, these gaps weaken the immediate translatability of current findings into universal sports nutrition products, underscoring the necessity for more rigorous and quantitative clinical research designs in the future.

## 10 Conclusion

Plant-derived bioactive peptides represent a highly promising and sustainable nutritional intervention for mitigating exercise-induced fatigue. Their multi-target efficacy—rooted in potent antioxidant, anti-inflammatory, and metabolic-regulatory properties—provides a distinct therapeutic advantage over conventional, single-target recovery supplements. Despite these remarkable capabilities, the translation from preclinical models to commercial sports nutrition is hindered by several critical bottlenecks. To maximize their applicability, specific directions for future research must be pursued. First, the microbiome-peptide interaction remains largely unexplored; future studies should investigate how specific gut microbiomes metabolize PBPs and whether this alters their bioavailability and efficacy. Second, computational biology and molecular docking studies are urgently needed to elucidate precise structure-activity relationships (SAR), which remain ambiguous. Finally, the field requires rigorous, large-scale human clinical trials to establish standardized dosing and timing protocols across diverse athletic populations. By addressing these unexplored dimensions, PBPs can seamlessly transition from promising bioactive compounds to foundational elements of evidence-based, personalized sports nutrition.
